# Multidrug-Resistant Gram-Negative Bacteria Contaminating Raw Meat Sold in Accra, Ghana

**DOI:** 10.3390/pathogens11121517

**Published:** 2022-12-11

**Authors:** Deric A. Baah, Fleischer C. N. Kotey, Nicholas T. K. D. Dayie, Francis S. Codjoe, Patience B. Tetteh-Quarcoo, Eric S. Donkor

**Affiliations:** 1 Department of Medical Microbiology, University of Ghana Medical School, Accra P.O. Box KB 4236, Ghana; 2 FleRhoLife Research Consult, Teshie, Accra P.O. Box TS 853, Ghana; 3 Department of Medical Laboratory Sciences, School of Biomedical & Allied Health Sciences, College of Health Sciences, University of Ghana, Accra P.O. Box KB 143, Ghana

**Keywords:** food safety, meat safety, antimicrobial resistance, multidrug resistance, extended-spectrum beta-lactamase, ESBL

## Abstract

Background: Efforts to combat antimicrobial resistance (AMR) should be based on the One Health approach, involving human health, animal health, and the environment. In Ghana, previous studies on AMR have given little attention to animal source food, a major route of transmission of antibiotic-resistant zoonotic pathogens. The aim of this study was to investigate the occurrence of multidrug-resistant (MDR) bacteria in meat sold in Accra. Methods: This was a cross-sectional study in which 270 meat samples (90 each of beef, goat meat, and chicken) were collected, and investigated for contamination with multidrug-resistant bacteria. The bacteria were subjected to susceptibility testing against amikacin (30 µg), ampicillin (10 µg), amoxicillin-clavulanate (20/10 µg), cefuroxime (30 µg), ceftriaxone (30 µg), ceftazidime (30 µg), cefepime (30 µg), ciprofloxacin (5 µg), trimethoprim-sulfamethoxazole (1.25/23.75 µg), ertapenem (10 µg), meropenem (10 µg), imipenem (10 µg), tigecycline (15 µg), and gentamicin (10 µg). Results: Thirty-two different types of bacteria, totalling 558, were isolated, the predominant being *Escherichia coli* (44.6%), *Aeromonas hydrophila* (19.9%), *Vibrio cholerae* (3.4%), *Aeromonas veronii* (3.2%), and *Klebsiella pneumoniae* (3.1%). The prevalence of MDR among the contaminating bacteria was 14.9%. The MDR distribution among the predominant bacteria was *Escherichia coli* (18.7%), *Aeromonas hydrophila* (11.1%), *Vibrio cholerae* and *Aeromonas veronii* (0.0% each), and *K. pneumoniae* (5.6%). Moreover, 2.0% of the contaminating bacteria were extended-spectrum beta-lactamase (ESBL) producers, all of which occurred in the chicken samples, and their distribution was: *Escherichia coli* (1.3%), *Klebsiella pneumoniae*, *Pantoea* spp., *Enterobacter cloacae*, and *Serratia plymuthica* (0.2% each). Conclusions: The meat samples were heavily contaminated with *Escherichia coli* and *Aeromonas hydrophila*, and less frequently, with *Vibrio cholerae*, *Klebsiella pneumoniae*, and other organisms. The prevalence of multidrug-resistant bacteria was moderate (14.9%), while that of ESBL producers was low (2%).

## 1. Introduction

The safety of meat and other animal source foods is under immense threat globally from chemicals and infectious agents [1,2,3,4]. Several studies conducted globally have identified multidrug-resistant (MDR) bacteria on meat products, some of which include methicillin-resistant *Staphylococcus aureus* (MRSA), extended-spectrum beta-lactamase (ESBL)-producing Enterobacteriaceae, MDR *Salmonella*, and MDR *Escherichia coli* 0157 [5,6,7,8,9]. Contaminated meats are one of the major causes of food-borne illnesses and an important conduit of zoonotic diseases [10]. Involvement of MDR organisms in the contamination could potentially exacerbate the already major menace of antimicrobial resistance (AMR), which is expected to culminate in an annual 10 million mortality rate and a loss of USD 100 trillion by 2050 should the problem not be effectively addressed [9,11,12,13,14].

The enormity and multifaceted nature of the AMR menace requires that remedial efforts be based on the One Health approach, involving human health, animal health, and the environment. Indeed, routine surveillance of AMR in animal source foods could serve as a “double-edged sword” that helps to address microbial food safety issues as well as the AMR menace. In Ghana, previous studies on AMR have given little attention to animal source food, which is a major route of transmission of antibiotic-resistant zoonotic pathogens [15,16,17,18,19,20,21,22,23,24,25,26,27,28]. Moreover, the wide usage of antibiotics in animal husbandry and the paradoxical paucity of AMR surveillance data regarding animal source foods means that data generated on AMR are far from robust [11,12,13,29]. This study was designed to help fill these knowledge gaps by determining the spectrum of bacterial pathogens contaminating three types of meat sold in Accra—beef, goat meat, and chicken—as well as the occurrence of multidrug resistance among the bacteria. This is especially important, as AMR in animal source foods needs continuous monitoring just as is being done in humans.

## 2. Materials and Methods

### 2.1. Study Site, Design and Sampling

**Figure 1:**
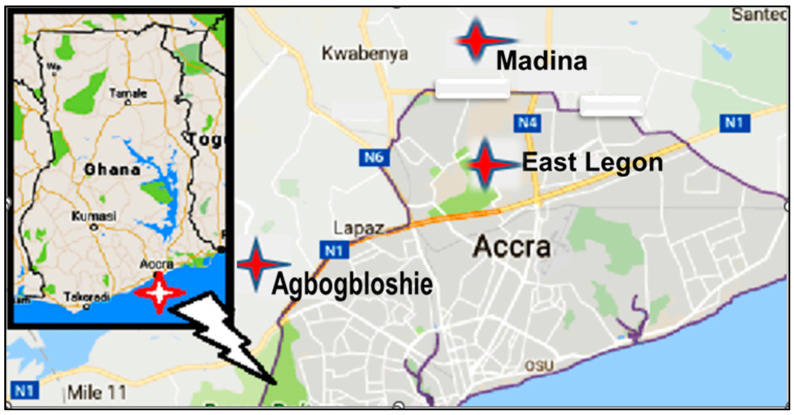
A map showing the study sites.

This was a cross-sectional study involving the collection of meat muscle samples (about 0.5 kg) from a variety of vending shops in Accra (which is the capital city of Ghana with a total land area covering 225.67 km^2^ and an estimated population of 1,665,086) [30]. In Accra, a greater number of butchers handle and process meats in ways that are inconsistent with microbiological food safety standards according to the Ghana Standards Authority and the Food and Drugs Authority [31]. Selected areas for the collection of the meat samples included meat vending shops at East Legon, Agbogbloshie Market, and Madina Market (Figure 1), where carcasses are purchased for subsequent processing and consumption in homes and eateries. In total, 270 meat samples were collected, evenly distributed across the three meat types—90 each of beef, goat meat, and chicken. Each meat sample, following its collection in the morning, was tightly sealed with sterile plastic wraps, placed in a cold box at 4 °C, and transported immediately to the research laboratory of the Department of Medical Microbiology at the University of Ghana Medical School, for microbiological analysis.

### 2.2. Laboratory Investigations

#### 2.2.1. Sample Preparation and Identification of Bacteria in the Meat Samples

Five to ten grams of each meat sample was mixed with 10 ml of peptone water (0.1%), and a homogenized suspension was prepared using sterile pestle and mortar. Homogenized suspensions were plated on MacConkey agar with crystal violet (Oxoid Ltd., Basingstoke, UK), blood agar (Oxoid Ltd., Basingstoke, UK), and xylose lysine deoxycholate (XLD) agar (Oxoid Ltd., Basingstoke, UK), and incubated aerobically for 16 to 18 h at 37 °C. The plates were subsequently inspected for growth, as well as for colonial morphology, such as lactose fermentation and hydrogen sulphide production. Subculturing was performed on MacConkey agar (Oxoid Ltd., Basingstoke, UK) with crystal violet and blood agar (Oxoid Ltd., Basingstoke, UK), following which the resultant pure cultures were initially identified by performing rapid indole and oxidase tests, followed by API 20E (Biomerieux SA, Marcy-l’Étoile, France), and MALDI-TOF (Bruker Daltonics GmbH & Co., Bremen, Germany, MBT Compass IVD Ver. 4.2.100).

For *Salmonella* isolates, serological identification was also employed, using the slide agglutination test. During the test, one drop (40 μL) of saline was placed on three separate glass slides. A portion of each isolate was emulsified in each saline drop, using a sterile inoculation loop to give a smooth, fairly dense suspension. To each slide bearing the emulsified suspension, one drop of either polyvalent O serum (Thermo Fisher Diagnostics BV, Landsmeer, The Netherlands), polyvalent H serum (Thermo Fisher Diagnostics BV, Landsmeer, The Netherlands), or polyvalent Vi serum (Thermo Fisher Diagnostics BV, Landsmeer, The Netherlands) was added. Each mixture was shaken for about three minutes, followed by observation for agglutination. *Salmonella* Typhimurium 4512:1:1, 2 NCTC 3048 was used as positive control, and *Hafnia alvei* NCTC 8535 was also used as negative control.

In the case of *Vibrio cholerae*, homogenized samples were cultured on thiosulphate citrate bile salt agar (TCBS) (Mast Group Ltd., Liverpool, Merseyside, UK) and inoculated in alkaline peptone water (Oxoid Ltd., Basingstoke, UK). TCBS culture plates were incubated at 16–18 h at 37 °C, and alkaline peptone water was incubated for 4 h at 37 °C and sub-cultured on 37 °C for 16–18 h. Identification of *Vibrio cholerae* was performed via Gram stain, indole and oxidase tests, and API 20E (Biomerieux SA, Marcy-l’Étoile, France). For serological identification, two separate drops (40 μL each) were placed on glass slides. The isolates were emulsified in each drop of saline to give a smooth, fairly dense suspension. To one suspension as a control, one drop (40 μL) of saline was added, and one drop (40 μL) of undiluted serum (*Vibrio cholerae* Remel^TM^, Dartford, Kent, UK) was added and mixed. The two slides were rocked for one minute and observed for agglutination by viewing against a dark background. Known positive and negative cultures were used as quality control checks.

Serological identification of species of *Shigella* was performed as follows: about 200 μL of saline was placed into a tube. Two colonies from overnight culture were emulsified in the saline to produce homogenized suspension. The latex reagents (Wellcolex^TM^ Colour *Shigella*, Thermo Fisher Diagnostics BV, Landsmeer, The Netherlands) were shaken intensely for a few seconds and dispensed into a separate circle on a flat reaction card. Using an applicator stick, the contents of each circle were mixed and spread to cover the whole area of the circle. The card was placed on a rotator and shook at 150 rpm for 2 min. The results were read using the Wellcolex* Colour *Shigella* Reading Guide.

#### 2.2.2. Antimicrobial Susceptibility Testing

Antibiotic susceptibility testing was performed according to guidelines set by the Clinical and Laboratory Standards Institute (CLSI) [32] using Kirby Bauer’s disc diffusion method on Mueller–Hinton agar (MHA) (Oxoid, Basingstoke, UK). Antibiotics discs, such as amikacin (30 μg), ampicillin (10 μg), ceftriaxone (30 μg), ceftazidime (30 µg) cefuroxime (30 μg), ciprofloxacin (10 μg), trimethoprim-sulfamethoxazole (1.25/23.75 μg), meropenem (10 μg), imipenem (10 μg), ertapenem (10 μg), tigecycline (15 μg), gentamicin (10 μg), cefepime (30 μg), and amoxicillin-clavulanate (20/10 μg) (Oxoid Ltd., Basingstoke, UK) were selected for testing. 

Pure colonies of isolates were inoculated in peptone water (Oxoid Ltd., Basingstoke, UK). The turbidity was adjusted to 0.5 McFarland standard using sterile peptone water and swabbed on MHA (Oxoid, Basingstoke, UK) in a manner allowing for semi-confluent growth post-incubation. Incubation was carried out at 37 °C for 24 h. After this, the inhibition zones were measured, and the results were interpreted using the CLSI [32] guidelines. Isolates showing resistance to three or more antibiotic classes were considered as MDR [33]. Moreover, the multiple antibiotic resistance index (MAR) was computed for each bacterial isolate as the fraction of the number of antibiotics to which an isolate displayed resistance out of the total number of antibiotics against which the susceptibility of the isolate was evaluated [34].

During the ESBL screening among the isolates, MHA (Oxoid, Basingstoke, UK) was inoculated with a standard inoculum (0.5 McFarland) of the test isolate, similar to the description in the preceding paragraph. Ceftazidime (30 μg) and ceftazidime-clavulanate (30 μg/10 μg) discs were placed on the surface of the agar and incubated at 37 °C for 16 to 18 h. Any bacterium in which an increase in zone diameter of ≥5 mm was observed after incubation in the presence of clavulanate than ceftazidime alone was determined to be an ESBL producer. *Escherichia coli* (*E. coli*) ATCC 25922 and *Klebsiella pneumoniae* (*K. pneumoniae*) ATCC 700603 were used as negative and positive control strains, respectively.

### 2.3. Data Analysis

The laboratory data collected were entered into STATA 14 (Strata Corp, College Station, TX, USA) for analysis. Descriptive statistics were used to summarize the data on the spectrum of bacterial pathogens contaminating the meat samples and their AMR rates, MDR prevalence, and MAR indices.

## 3. Results

### 3.1. Spectrum of Bacterial Pathogens Contaminating the Meats

All the individual meat samples had bacterial contamination—5.9% (*n* = 16) with one bacterium, 83.3% (*n* = 225) with two bacteria, 8.9% (*n* = 24) with three bacteria, and 1.9% (*n* = 5) with four bacteria. A summary of the number of individual bacteria isolated per sample is presented in Table 1.

The spectrum of the bacterial contaminants was broad, involving 32 different types of bacteria totalling 558. The predominant ones were *E. coli* [262; beef = 30.5%, *n* = 80; goat meat = 30.5%, *n* = 80; chicken = 38.9%, *n* = 102], *Aeromonas hydrophila* (*A. hydrophila*) [117; beef = 35.9%, *n* = 42; goat meat = 53.0%, *n* = 62; chicken = 11.1%, *n* = 13], *Vibrio cholerae* (*V. cholerae*) [20; beef = 50.0%, *n* = 10; goat meat = 50.0%, *n* = 10; chicken = 0.0%, *n* = 0], *Aeromonas veronii* (*A. veronii*) [19; beef = 63.1%, *n* = 12; goat meat = 36.8%, *n* = 7; chicken = 0.0%, *n* = 0], and *K. pneumoniae* [18; beef = 22.2%, *n* = 4; goat meat = 16.7%, *n* = 3; chicken = 61.1%, *n* = 11]. The distribution of the bacterial contaminants is presented in Table 2.

### 3.2. Antimicrobial Resistance among the Bacterial Contaminants of the Meats

Almost all the meat samples (96.7%; *n* = 261) were contaminated with antibiotic-resistant bacteria—beef (97.8%; *n* = 88), goat (97.8%; *n* = 88), and chicken (94.4%; *n* = 85). When the resistance data for all the bacteria are put together, the highest resistance rate was recorded against ampicillin (83.3%), followed by amoxicillin-clavulanate (36%). The rates of cefuroxime, ceftriaxone, ceftazidime, cefepime, ciprofloxacin, trimethoprim-sulphamethoxazole, ertapenem, meropenem, and tetracycline ranged between 1.3% and 19.5%, whereas no resistance was recorded against either of amikacin, imipenem, and gentamicin for any of the bacterial contaminants. In *E. coli*, the antibiotics whose resistance rates were the highest were ampicillin (81.3%), trimethoprim-sulphamethoxazole (26.3%), and amoxicillin-clavulanate (25.2%). With regard to *A. hydrophila*, the antibiotics whose resistance rates were the highest were ampicillin (94%), amoxicillin-clavulanate (62.4%), and cefuroxime (10.3%). As regards *V. cholerae*, the antibiotics whose resistance rates were the highest were ampicillin (90%) and trimethoprim-sulphamethoxazole (25%), with no resistance recorded against any of the remaining antibiotics that were tested. In *A. veronii*, all the organisms were resistant to ampicillin and amoxicillin-clavulanate but not to any of the remaining antibiotics that were tested; these rates were identical, regardless of whether the meat type was beef, goat, or chicken. With respect to *K. pneumoniae*, the antibiotics whose resistance rates were the highest were ampicillin (94.4%) and trimethoprim-sulphamethoxazole (27.8%). Details of the AMR rates of the bacterial contaminants are presented in Table 3.

As observed in Table 4, the prevalence of MDR among the contaminating bacteria was 14.9% (*n* = 83)—11.3% (*n* = 21) in beef, 14.7% (*n* = 28) in goat meat, and 18.8% (*n* = 34) in chicken. Additionally, the MDR distribution among the predominant bacteria was *E. coli* (18.7%, *n* = 49), *A. hydrophila* (11.1%, *n* = 13), *V. cholerae* and *A. veronii* (0.0% each), and *K. pneumoniae* (5.6%, *n* = 1) (Table 4). Moreover, the mean MAR index, as a composite, was 0.12 ± 0.09 (beef = 0.11 ± 0.08; goat meat = 0.11 ± 0.07; chicken = 0.13 ± 0.12), with 15.23% (*n* = 85) (beef = 10.8% [*n* = 20]; goat meat = 15.71% [*n* = 30]; chicken = 19.33% [*n* = 35]) of the bacteria recording a MAR index greater than 0.2 (Table 4). Additionally, 2.0% (*n* = 11) of the contaminating bacteria were ESBL producers, all of which occurred in 11 of the chicken samples, and their distribution was: *E. coli* (1.3%, *n* = 7), *K. pneumoniae*, *Pantoea* spp., *Enterobacter cloacae*, and *Serratia plymuthica* (0.2% each, *n* = 1).

## 4. Discussion

The purpose of the current study was to investigate the occurrence of multidrug-resistant bacteria in meats (beef, goat meat, and chicken) sold in Accra. One major focus of it was to determine the spectrum of bacteria contaminating the meat samples. The bacterial contaminants were observed to be of a broad range, involving 32 different types totalling 558, with the predominant ones being *E. coli*, *A. hydrophila*, *V. cholerae*, *A. veronii*, and *K. pneumoniae*. In the study of Addo et al. [35], the predominant bacterial contaminant was *E. coli* (69.76%) as well, and other recovered bacterial contaminants comprised *Klebsiella* spp., *Aeromonas spp*., *Pseudomonas aeruginosa*, *Enterobacter* spp., *Bacillus* spp., *Yersinia enterocolitica*, *Listeria monocytogenes*, *Salmonella* Typhimurium, and *Staphylococcus aureus*. Adzitey et al. [36] and Adzitey et al. [37], whose studies were conducted on various meat types in the Tamale Metropolis, also reported *E. coli* at rates of 84–100%. In a study carried out by Yar et al. [38] that evaluated the microbiological quality of chicken imported from Brazil, Netherlands, and the USA, and sold in the Kumasi Metropolis as well, the researchers reported a mixture of bacteria and fungi contaminating the meat samples with the following distribution: bacteria (*Klebsiella* spp. [13.8%], *Salmonella* spp. [24.6%], *E. coli* [26.2%], and *Staphylococcus* spp. [35.4%]) and fungi (*Cladosporium* spp. [15.2%], *Penicillium* spp. [24.2%], *Rhizopus* spp. [27.3%], *Aspergillus* spp. [33.3%]). Contrastingly, Dsani et al. [39] whose study was on a mixture of 205 carcasses of mutton (*n* = 16), chevon (*n* = 108), and beef (*n* = 81), and focused on *E. coli* reported an *E. coli* prevalence of 48%.

The high diversity of the bacterial contaminants is a reflection of the wide range of infections to which consumers of the meats predispose themselves, especially if those meats are not well-cooked prior to their consumption. Contamination of the meats with zoonotic pathogens, such as *Salmonella* spp., *Yersinia enterolitica*, and *Shigella flexneri*, also indicates a potential for zoonotic transmission of these pathogens to the consumers of the meats, although this risk cannot be accurately quantified with the available data. Moreover, the high bacterial contamination rate of the various meat types, particularly, with *E. coli*, is of grave concern, as *E. coli* presence indicates faecal contamination of the meats, and could potentially result in gastroenteritis among the consumers [40,41].

That said, the high bacterial contamination rate is not surprising, as the hygienic practices of the vendors were generally poor. For example, the following were common practice among the vendors: chatting with each other, open display of carcasses without covers, use of unsterilized knives and other cutting edges for butchering the meats, inadequate control of insects, such as houseflies, and sneezing or coughing during meat handling, situations prevalent in many market settings in the country. Adzitey et al. [42] whose study was conducted in the Tamale Metropolis on mutton and chevon reported similar observations. Interestingly, Koffi-Nevry et al. [43], who carried out their study in Cote D’Ivoire echoed similar observations, which suggests that poor meat handling practices may be a widespread problem. Consequently, it would be necessary for regulatory bodies in the country to increase the robustness with which they monitor and enforce the microbial safety of meats and other foods, as well strict adherence of vendors to good food handling practices. It would also be necessary to concurrently sensitize consumers on the need to ensure that they cook their meats well to allow for removal of contaminating bacteria before they consume them.

It is noted that the bacterial isolates from the meat samples showed high-level resistance to ampicillin (83.3%), amoxicillin-clavulanate (36%) and trimethoprim-sulphamethoxazole (19%). The high rates of resistance of greater than 50% to penicillin and its derivatives from meat have been reported by several studies in Ghana [37,39,44]. In Dsani et al.’s [39] study conducted in Accra, 57% of the bacterial isolates from animal source foods showed resistance to ampicillin. Another study performed in Ghana showed a 70.97% resistance to amoxicillin-clavulanic acid combination [44]. The high level of resistance to ampicillin (a derivative of penicillin) may be due to easy access to penicillin and its frequent use in animal husbandry. The high rates of susceptibility of the bacterial isolates to meropenem (100%), ertapenem (98.72), imipenem (100%), amikacin (100%), and tigecycline (100%) could be explained by their non-routine use in animal husbandry. Similar to this observation, in the study of Dsani et al. [39], the reported *E. coli* resistance rates increased across trimethoprim-sulphamethoxazole (17%), cefuroxime (21%), tetracycline (45%), and ampicillin (57%), and the susceptibility rates increased across amikacin (92%), ciprofloxacin (92%), gentamicin (97%), chloramphenicol (97%), cefotaxime (98%), and ceftriaxone (99%), and no meropenem resistance was recorded. Moreover, Adzitey et al. [37] reported *E. coli* resistance rates of ampicillin (71.67%), tetracycline (73.33%), and erythromycin (85.00%).

In this study, the prevalence of MDR and ESBLs among the contaminating bacteria was 14.9% and 2%, respectively, with a good proportion of the contaminants (15.23) recording an MAR index of greater than 0.2. The highest occurrence of MDR organisms was observed in chicken, with the highest magnitude of occurrence seen in isolates of *E. coli.* Dsani et al. [39], cited earlier, reported their MDR prevalence to be 22.4%, and that of ESBL producers to be 14.3%. Adzitey et al. [37] reported a higher MDR prevalence in their study (68.33%), but ESBL production was not reported. It is noted that many studies of this nature on occurrence have yielded different results, but higher rates of resistance are observed in chicken. In a recent study involving only *E. coli* isolates, the prevalence of MDR in meat samples was reported to be 22% and the highest occurrence was seen in isolates from chicken. In a study conducted in Nepal, the prevalence of MDR organisms in the meat samples was reported to be 32.7%, with the highest occurrence observed in bacteria isolates from chicken relative to buffalo meat [45]. The high occurrence of MDR organisms in chicken can be due to the routine use of antibiotics in poultry, not only therapeutically, but also for growth promotion [46]. Generally, the presence of antibiotic-resistant organisms in meats usually demonstrate the resistant situations in the gut of the animals and environments in which the animals are slaughtered and handled [47].

MDR is a major public health and economic concern across the globe. The presence of MDR in the meat-related bacteria observed in the current study is a cause for worry, for several reasons. First, poor cooking of such meats could predispose consumers to not just colonization with these MDR pathogens, but also infections with them. Under such circumstances, the potential for dissemination of the resistance traits from the MDR organisms to the microbiota with whom they co-colonize the gut cannot be overlooked [48]. In like manner, it is possible for the resistance traits to be transferred to other organisms in circulation when colonized persons shed them in faecal matter. It will not be far-fetched to consider the possibility of MDR bacteria spreading from meat markets to hospital environments via rodents, cockroaches and other insects, and other vehicles for transmission of food-borne pathogens [49,50,51,52]. The risk of transmission could especially be pronounced in cases of close proximity between markets and healthcare facilities. In hospital settings, these pathogens could be disseminated further and potentially negatively impact disease outcomes of patients, as well as healthcare costs [49,53,54,55,56].

One limitation of the study is that the loads of the bacteria in the meat samples were not determined. Moreover, owing to the choice of culture media and incubation conditions, it is possible that anaerobic and microaerophilic bacteria that may have been present in the meat samples were missed.

## 5. Conclusions

The meat samples were heavily contaminated with *Escherichia coli* and *Aeromonas hydrophila*, and less frequently, with *Vibrio cholerae*, *Klebsiella pneumoniae*, and other organisms. The prevalence of multidrug resistant bacteria was moderate (14.9%), while that of ESBL producers was low (2%).

The high prevalence of *E. coli*, a key indicator of faecal contamination, in the meats, suggests that the slaughterers and meat vendors need to be sound education on good food handling practices. Furthermore, studies of this nature need to be routinely conducted to fill gaps on AMR surveillance. These studies could expand the range of culture media and incubation conditions to detect the possible presence of anaerobic and microaerophilic food-borne bacterial pathogens.

## Figures and Tables

**Table 1:** A summary of the number of individual bacteria isolated per sample.

Number of Individual Bacterial Contaminants Per Sample	Number of Samples			
	All Meat Types	Beef	Goat	Chicken
One	16 (5.9%)	0 (0.0%)	1 (1.1%)	15 (16.7%)
Two	225 (83.3%)	85 (94.4%)	77 (85.6%)	63 (70.0%)
Three	24 (8.9%)	4 (4.4%)	12 (13.3%)	8 (8.9%)
Four	5 (1.9%)	1 (1.1%)	0 (0.0%)	4 (4.4%)

**Table 2:** Distribution of the bacteria contaminating the meat sold in the markets.

Isolated Bacterium	Total Number (*n*, %) *	Distribution of the Bacterium Across the Meat Types ^#^		
		Beef (*n*, %)	Goat (*n*, %)	Chicken (*n*, %)
*Escherichia coli*	262 (47.0%)	80 (30.5%)	80 (30.5%)	102 (38.9%)
*Aeromonas hydrophila*	117 (21.0%)	42 (35.9%)	62 (53.0%)	13 (11.1%)
*Vibrio cholerae*	20 (3.6%)	10 (50.0%)	10 (50.0%)	0 (0.0%)
*Aeromonas veronii*	19 (3.4%)	12 (63.1%)	7 (36.8%)	0 (0.0%)
*Klebsiella pneumoniae*	18 (3.2%)	4 (22.2%)	3 (16.7%)	11 (61.1%)
*Serratia plymuthica*	14 (2.5%)	1 (7.1%)	2 (14.3%)	11 (78.6%)
*Pantoea* spp.	13 (2.3%)	1 (7.7%)	1 (7.7%)	11 (84.6%)
*Moellerella wisconsensis*	10 (1.8%)	7 (70.0%)	3 (30.0%)	0 (0.0%)
*Acinetobacter baumannii*	9 (1.6%)	1 (11.1%)	1 (11.1%)	7 (77.8%)
*Vibrio* spp.	9 (1.6%)	4 (44.4%)	4 (44.4%)	1 (11.1%)
*Enterobacter cloacae*	7 (1.3%)	2 (28.6%)	0 (0.0%)	5 (71.4%)
*Vibrio alginolyticus*	6 (1.1%)	1 (16.7%)	3 (50.0%)	2 (33.3%)
*Pseudomonas luteola*	6 (1.1%)	3 (50.0%)	3 (50.0%)	0 (0.0%)
*Proteus mirabilis*	6 (1.1%)	4 (66.7%)	0 (0.0%)	2 (33.3%)
*Salmonella enteritidis*	6 (1.1%)	2 (33.3%)	2 (33.3%)	2 (33.3%)
*Citrobacter koseri*	5 (0.9%)	3 (60.0%)	1 (20.0%)	1 (20.0%)
*Yersinia enterolytica*	5 (0.9%)	0 (0.0%)	0 (0.0%)	5 (100.0%)
*Shigella flexneri*	3 (0.5%)	0 (0.0%)	0 (0.0%)	3 (100.0%)
*Enterobacter aerogenes*	3 (0.5%)	2 (66.7%)	1 (33.3%)	0 (0.0%)
*Citrobacter freundii*	3 (0.5%)	2 (66.7%)	1 (33.3%)	0 (0.0%)
*Rahnella aqualitis*	2 (0.4%)	0 (0.0%)	0 (0.0%)	2 (100.0%)
*Serratia odorifera*	2 (0.4%)	0 (0.0%)	0 (0.0%)	2 (100.0%)
*Citrobacter youngae*	2 (0.4%)	1 (50.0%)	1 (50.0%)	0 (0.0%)
*Klebsiella oxytoca*	2 (0.4%)	2 (100.0%)	0 (0.0%)	0 (0.0%)
*Providencia rettgeri*	2 (0.4%)	1 (50.0%)	1 (50.0%)	0 (0.0%)
*Acinetobacter iwoffi*	1 (0.2%)	1 (100.0%)	0 (0.0%)	0 (0.0%)
*Serratia rubidaea*	1 (0.2%)	1 (100.0%)	0 (0.0%)	0 (0.0%)
*Kluyvera* spp.	1 (0.2%)	0 (0.0%)	1 (100.0%)	0 (0.0%)
*Pasteurella multocida*	1 (0.2%)	0 (0.0%)	1 (100.0%)	0 (0.0%)
*Yersinia ruckeri*	1 (0.2%)	0 (0.0%)	1 (100.0%)	0 (0.0%)
*Stenotrophomonas maltophilia*	1 (0.2%)	0 (0.0%)	1 (100.0%)	0 (0.0%)
*Pasteurella pneumotropica*	1 (0.2%)	0 (0.0%)	0 (0.0%)	1 (100.0%)
Total	558 (100%)	186 (33.3%)	191 (34.2%)	181 (32.4%)

* The proportions were computed using the total number of bacteria as denominator; ^#^ The proportions were computed using the total number of each bacterium as denominator.

**Table 3:** Antimicrobial resistance rates of the bacterial species contaminating the meat samples.

Organisms/Antibiotics	AMP	AMC	CEFU	CEFT	CFTZ	CEFP	CIP	TMS	ERT	MEM
All Bacteria (*n* = 558)	83.3	36	9.1	2.2	2.2	2.3	5.7	19.5	1.3	1.3
All *E. coli* (*n* = 262)	81.3	25.2	7.6	2.7	2.7	3.1	9.5	26.3	1.1	1.1
Beef *E. coli* (*n* = 80)	78.8	42.5	3.8	0	0	1.3	10	28.8	1.3	1.3
Goat *E. coli* (*n* = 80)	77.5	23.8	6.3	0	0	0	11.3	12.5	0	0
Chicken *E. coli* (*n* = 102)	86.3	12.7	11.8	6.9	6.9	6.9	7.8	26.5	2	2
All *A. hydrophila* (*n* = 117)	94	62.4	10.3	0	0	0	3.4	6	0	0
Beef *A. hydrophila* (*n* = 42)	100	88.1	2.4	0	0	0	3.2	1.6	0	0
Goat *A. hydrophila* (*n* = 62)	88.7	53.2	17.7	0	0	0	3.2	3.2	0	0
Beef *A. hydrophila* (*n* = 13)	100	23.1	0	0	0	0	0	30.8	0	0
All *V. cholerae* (*n* = 20)	90	0	0	0	0	0	0	25	0	0
Beef *V. cholerae* (*n* = 10)	100	0	0	0	0	0	0	0	0	0
Goat *V. cholerae* (*n* = 10)	80	0	0	0	0	0	0	50	0	0
All *A. veronii* (*n* = 19)	100	100	0	0	0	0	0	0	0	0
Beef *A. veronii* (*n* = 12)	100	100	0	0	0	0	0	0	0	0
Goat *A. veronii* (*n* = 7)	100	100	0	0	0	0	0	0	0	0
All *K. pneumoniae* (*n* = 18)	94.4	11.1	5.6	5.6	5.6	5.6	0	27.8	0	0
Beef *K. pneumoniae* (*n* = 4)	75	0	0	0	0	0	0	25	0	0
Goat *K. pneumoniae* (*n* = 3)	100	0	0	0	0	0	0	66.7	0	0
Chicken *K. pneumoniae* (*n* = 11)	100	18.2	9.1	9.1	9.1	9.1	0	18.2	0	0

Amikacin, imipenem, tigecycline, and gentamicin are not included in the table, due to absence of resistance to any of them; AMP = Ampicillin; AMC = Amoxicillin-clavulanate; CEFU = Cefuroxime; CEFT = Ceftriaxone; CFTZ = Ceftazidime; CEFP = Cefepime; CIP = Ciprofloxacin; TMS = Trimethoprim-sulphamethoxazole; ERT = Ertapenem; MEM = Meropenem.

**Table 4:** Multidrug resistance among the bacterial contaminants.

Bacteria/Meat Types	MDR Prevalence	MAR Index
All Bacteria (from all meat types) (*n* = 558)	14.9% (*n* = 83)	0.12 ± 0.09
All Bacteria from Beef (*n* = 186)	11.3% (*n* = 21)	0.11 ± 0.08
All Bacteria from Goat (*n* = 191)	14.7% (*n* = 28)	0.11 ± 0.07
All Bacteria from Chicken (*n* = 181)	18.8% (*n* = 34)	0.13 ± 0.12
All *E. coli* (*n* = 262)	18.7% (*n* = 49)	0.11 ± 0.10
Beef *E. coli* (*n* = 80)	18.8% (*n* = 15)	0.12 ± 0.09
Goat *E. coli* (*n* = 80)	18.8% (*n* = 15)	0.10 ± 0.08
Chicken *E. coli* (*n* = 102)	18.6% (*n* = 19)	0.12 ± 0.12
All *A. hydrophilia* (*n* = 117)	11.1% (*n* = 13)	0.13 ± 0.05
Beef *A. hydrophilia* (*n* = 42)	7.1% (*n* = 3)	0.14 ± 0.03
Goat *A. hydrophilia* (*n* = 62)	14.5% (*n* = 9)	0.12 ± 0.06
Chicken *A. hydrophilia* (*n* = 13)	7.7% (*n* = 1)	0.11 ± 0.05
All *V. cholerae* (*n* = 20)	0.0% (*n* = 0)	0.08 ± 0.04
Beef *V. cholerae* (*n* = 10)	0.0% (*n* = 0)	0.09 ± 0.03
Goat *V. cholerae* (*n* = 10)	0.0% (*n* = 0)	0.08 ± 0.05
All *A. veronii* (*n* = 19)	0.0% (*n* = 0)	0.14 ± 0.00
Beef *A. veronii* (*n* = 12)	0.0% (*n* = 0)	0.14 ± 0.00
Goat *A. veronii* (*n* = 7)	0.0% (*n* = 0)	0.14 ± 0.00
All *K. pneumoniae* (*n* = 18)	5.6% (*n* = 1)	0.12 ± 0.10
Beef *K. pneumoniae* (*n* = 4)	0.0% (*n* = 0)	0.11 ± 0.04
Goat *K. pneumoniae* (*n* = 3)	0.0% (*n* = 0)	0.12 ± 0.04
Chicken *K. pneumoniae* (*n* = 11)	9.1% (*n* = 1)	0.12 ± 0.13

MDR = Multidrug resistance; MAR = Multiple antibiotic resistance index.

## Data Availability

The data presented in this study are available upon reasonable request from the corresponding author via esampane-donkor@ug.edu.gh.
